# A Change

**Published:** 1893-05

**Authors:** 


### A Change.

During the latter part of March last, 1893, quite a change was effected in the control and work of the Dental Department of the Cincinnati College of Medicine and Surgery. The dental members of the faculty of the department all tendered their resignations, which were accepted, and on the same evening the places were filled by the appointment of Dr. J. R. Callahan, as Dean and Professor of Prosthetic Dentistry; Dr. M. H. Fletcher, Professor of Physiology, Histology and Operative Dentistry; and Dr. A. O. Heise, as Professor of Dental Pathology and Materia Medica.

The new faculty began their work on the morning following their election, and carried the course of instruction through to its end. An account of the commencement will be found in this number.

We have no doubt that in the hands of these teachers thorough, efficient and acceptable work will be done, which is the great desideratum in dental college work. The reputation and standing of every college should be based upon the efficiency of its work, and if this one takes a high standing long may it live.

